# Supplementary material for: Synthesis, Molecular Docking Analysis, and Carbonic Anhydrase Inhibitory Evaluations of Benzenesulfonamide Derivatives Containing Thiazolidinone
Source: Molecules. 2019 Jun 30;24(13):2418. doi: 10.3390/molecules24132418 (PMC6651801; doi:10.3390/molecules24132418)
Supplement: Supplementary file 1 [file molecules-24-02418-s001.pdf]

Supporting Information: Synthesis, molecular docking analysis, and carbonic anhydrase IX inhibitory evaluations of benzenesulfonamide derivatives containing thiazolidinone

Zuo-Peng Zhang, Ze-Fa Yin, Jia-Yue Li, Zhi-Peng Wang, Qian-Jie Wu, Jian Wang, Yang Liu*, Mao-Sheng Cheng*
Key Laboratory of Structure-Based Drug Design & Discovery of Ministry of Education, School of Pharmaceutical Engineering, Shenyang Pharmaceutical University, Shenyang 110016, PR China.
* Corresponding authors. Email: y.liu@syphu.edu.cn (Y. Liu); mscheng@syphu.edu.cn (M. Cheng)

Table S1. Crystal data and structure refinement for compound 13.

Identification code	ZZP1112
Empirical formula	$C_{26}H_{25}N_3O_3S_2$
Formula weight	491.61
Temperature/K	100.00(10)
Crystal system	triclinic
Space group	P-1
a/Å	11.3246(7)
b/Å	11.3412(8)
c/Å	12.4507(8)
α/°	66.290(7)
β/°	89.665(5)
γ/°	67.305(6)
Volume/Å^3	1329.49(17)
Z	2
ρ_{calc}g/cm^3	1.228
μ/mm^{-1}	0.231
F(000)	516.0
Crystal size/mm^3	0.13 × 0.12 × 0.11
Radiation	MoKα (λ = 0.71073)
2Θ range for data collection/°	3.96 to 58.972
Index ranges	-15 ≤ h ≤ 14, -14 ≤ k ≤ 15, -16 ≤ l ≤ 16
Reflections collected	17723
Independent reflections	6410 [R_{int} = 0.0362, R_{sigma} = 0.0469]
Data/restraints/parameters	6410/9/316
Goodness-of-fit on F^2	1.077
Final R indexes [I>=2σ (I)]	R_1 = 0.0645, wR_2 = 0.1657
Final R indexes [all data]	R_1 = 0.0773, wR_2 = 0.1765
Largest diff. peak/hole / e Å^{-3}	0.72/-0.68

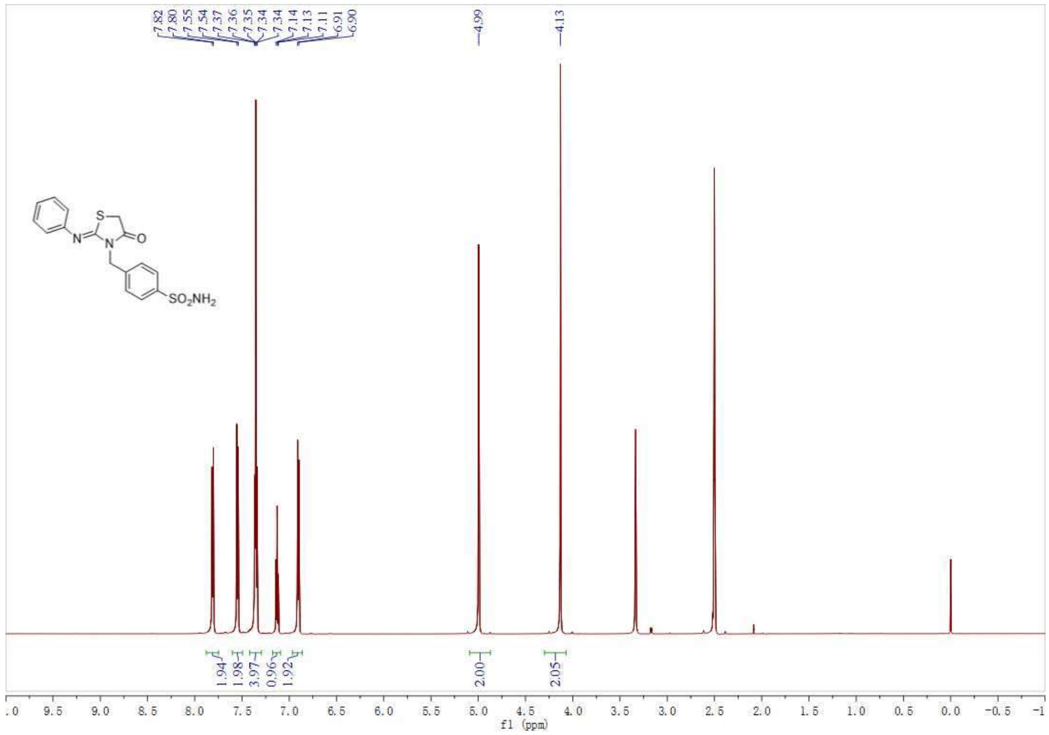

Figure S1. The ^{1}H-NMR of compound 2

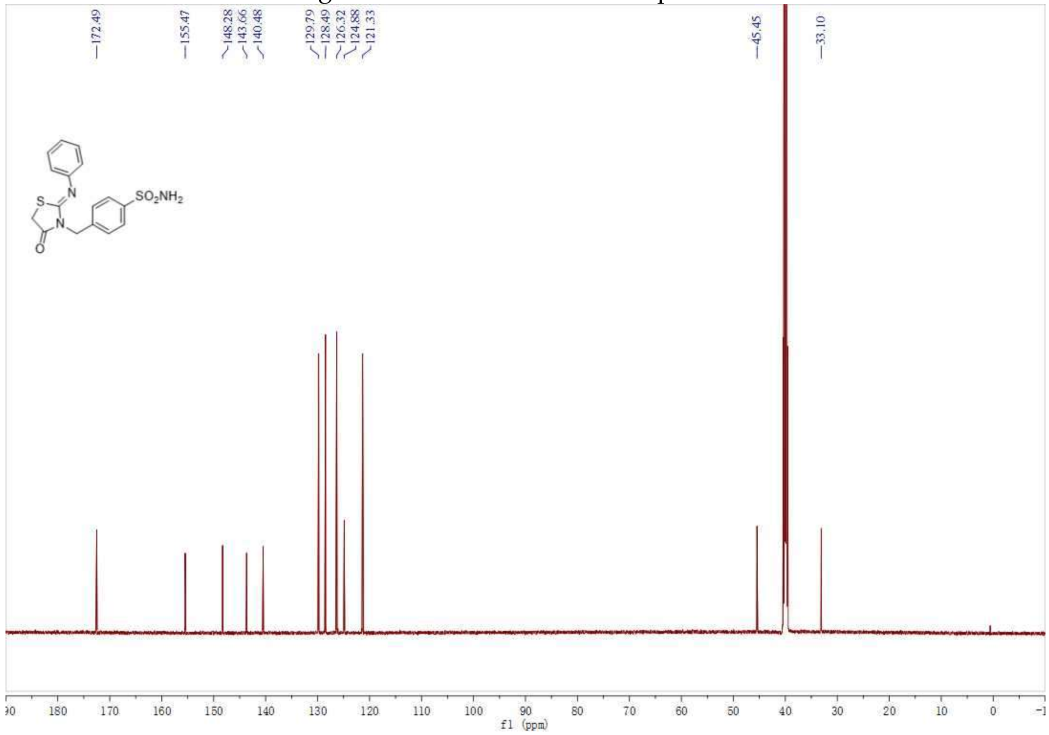

Figure S2. The ^{13}C-NMR of compound 2

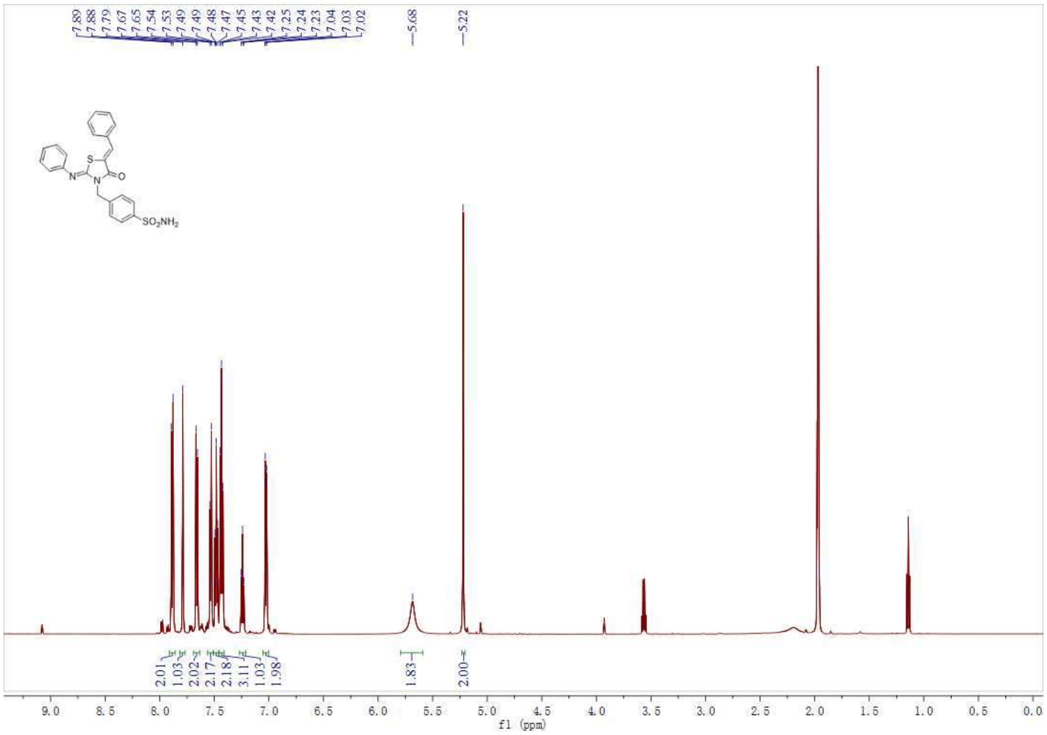

Figure S3. The ^{1}H-NMR of compound 3

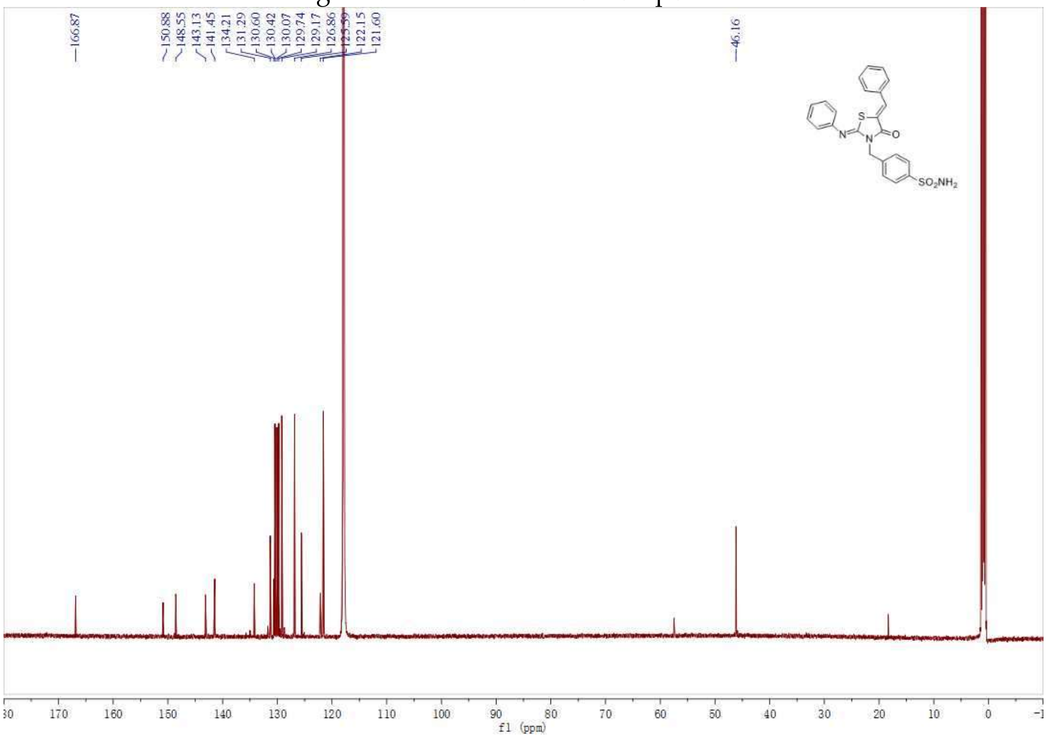

Figure S4. The ^{13}C-NMR of compound 3

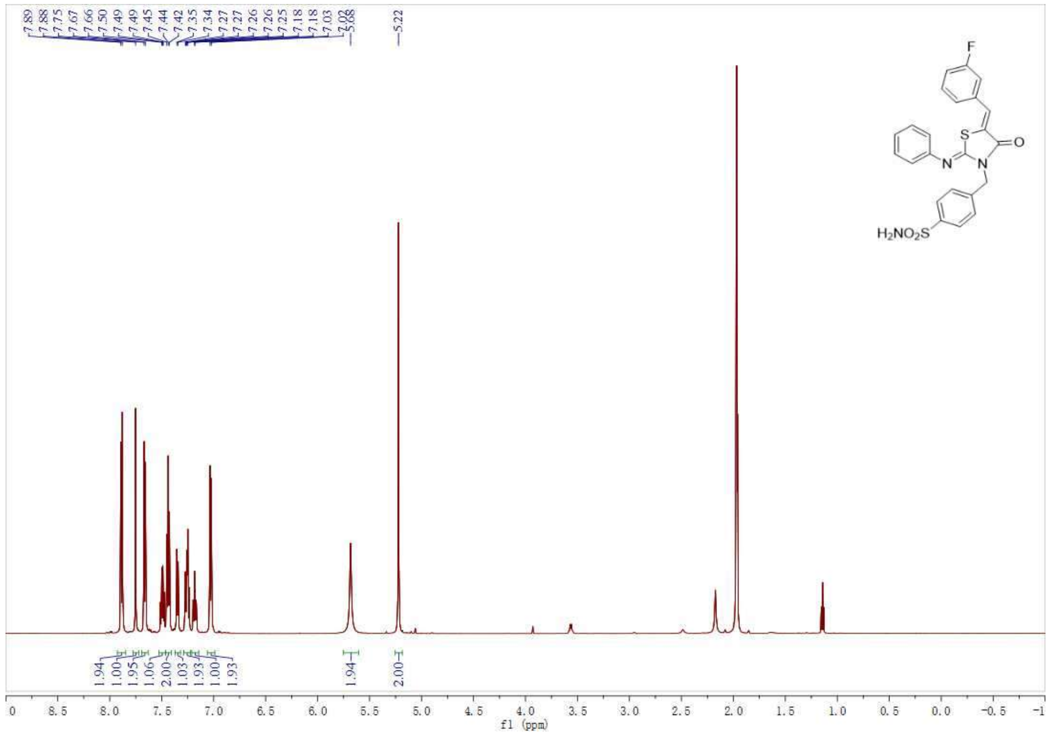

Figure S5. The ^{1}H-NMR of compound 4

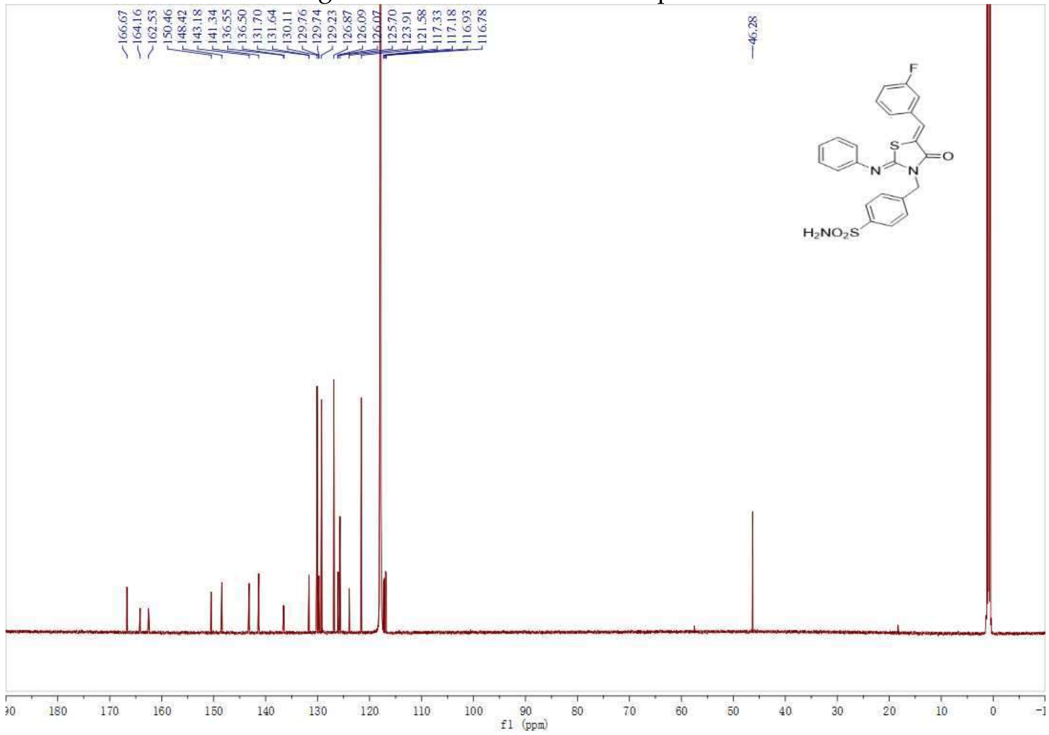

Figure S6. The ^{13}C-NMR of compound 4

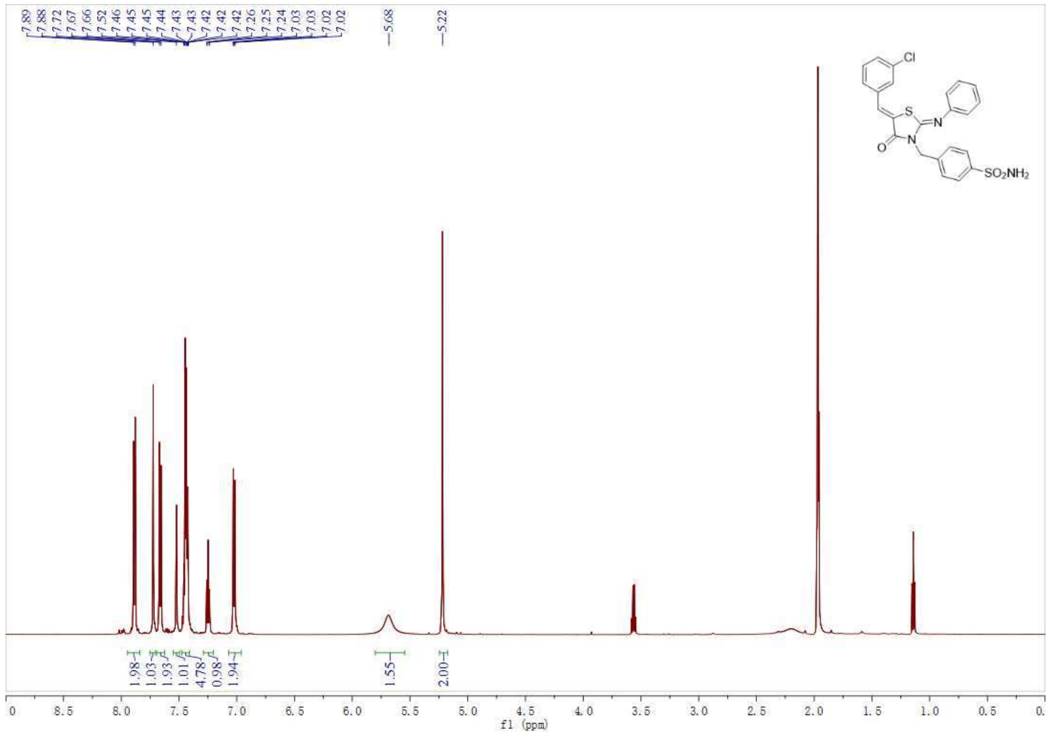

Figure S7. The ^{1}H-NMR of compound 5

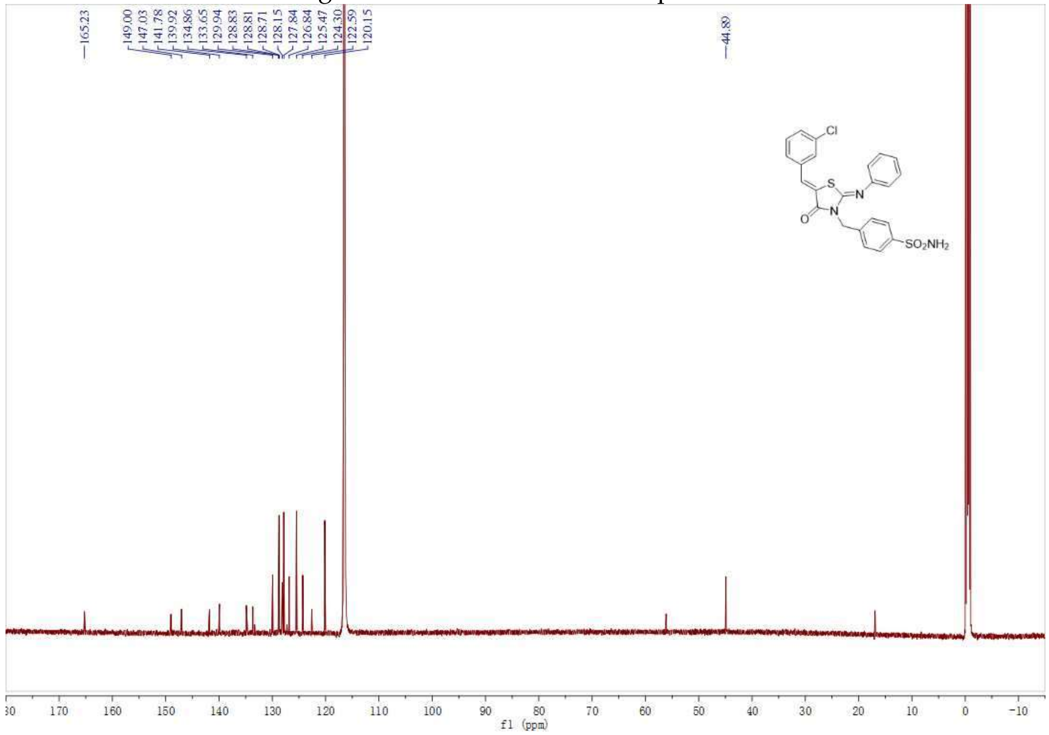

Figure S8. The ^{13}C-NMR of compound 5

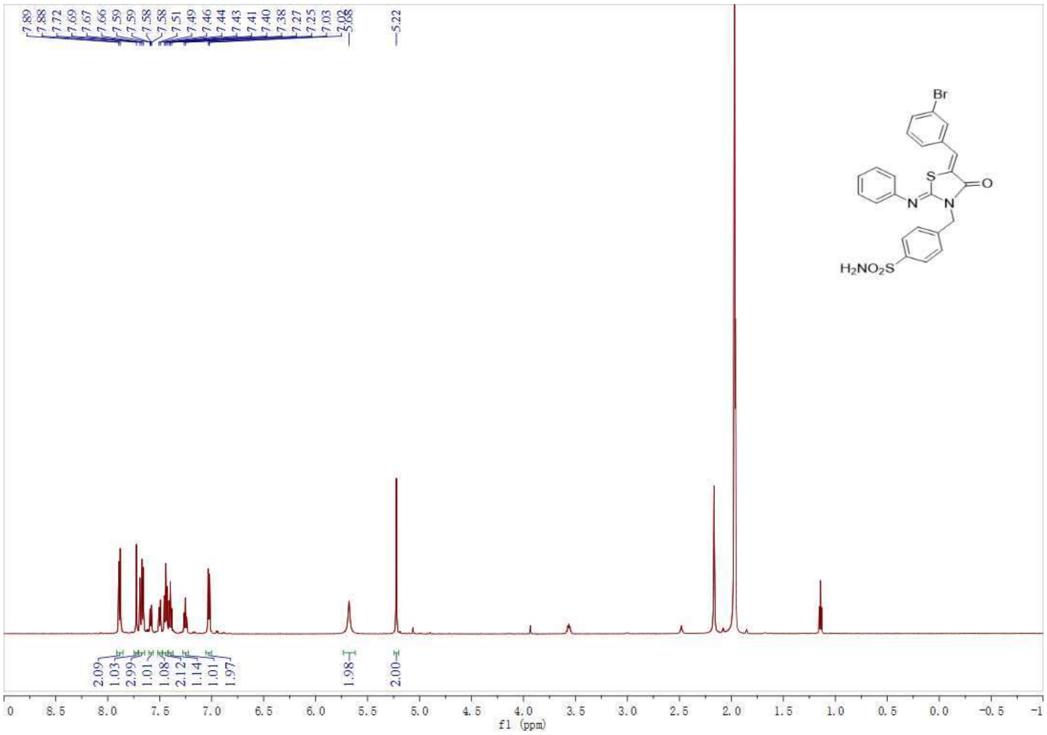

Figure S9. The ^{1}H-NMR of compound 6

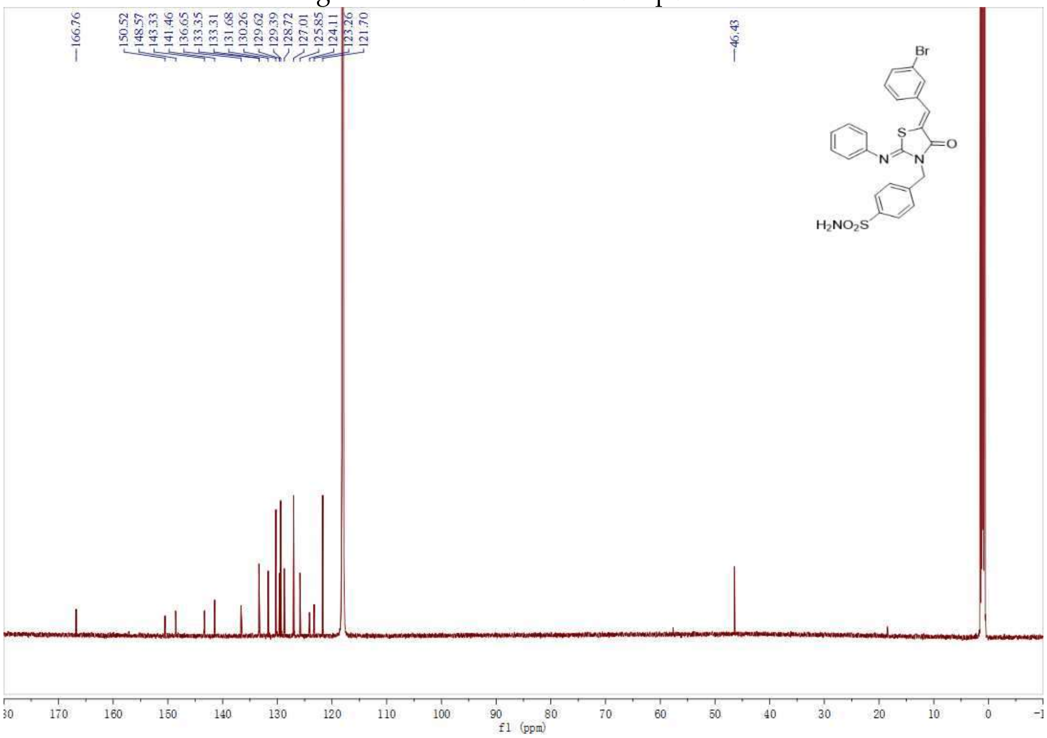

Figure S10. The ^{13}C-NMR of compound 6

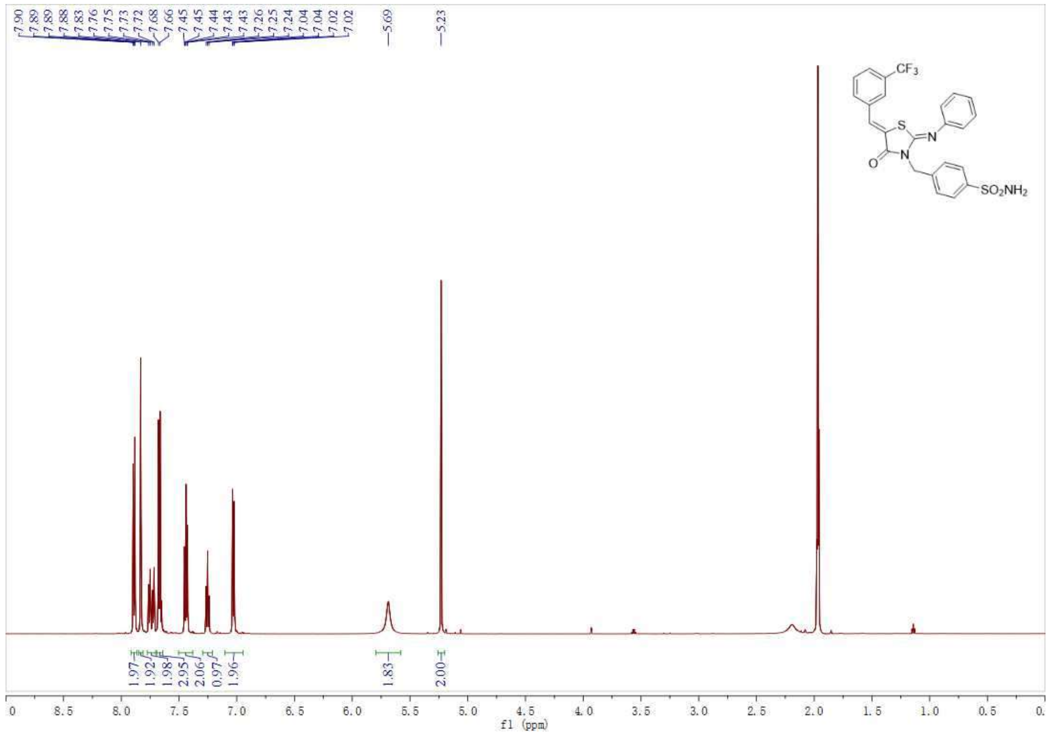

Figure S11. The ^{1}H-NMR of compound 7

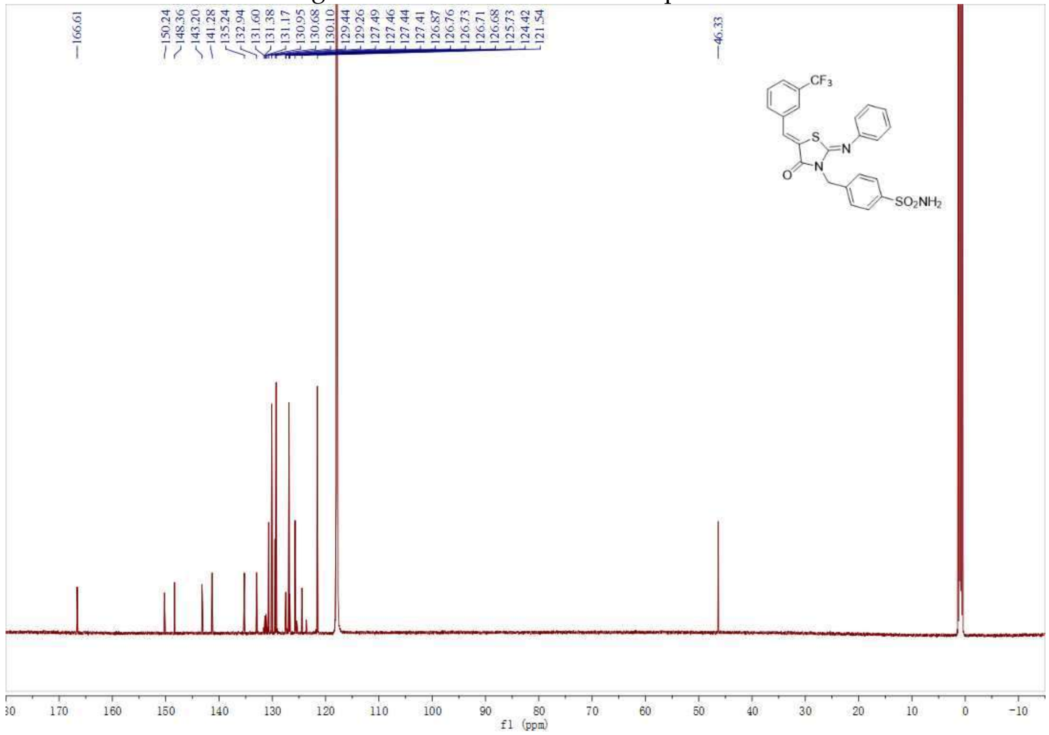

Figure S12. The ^{13}C-NMR of compound 7

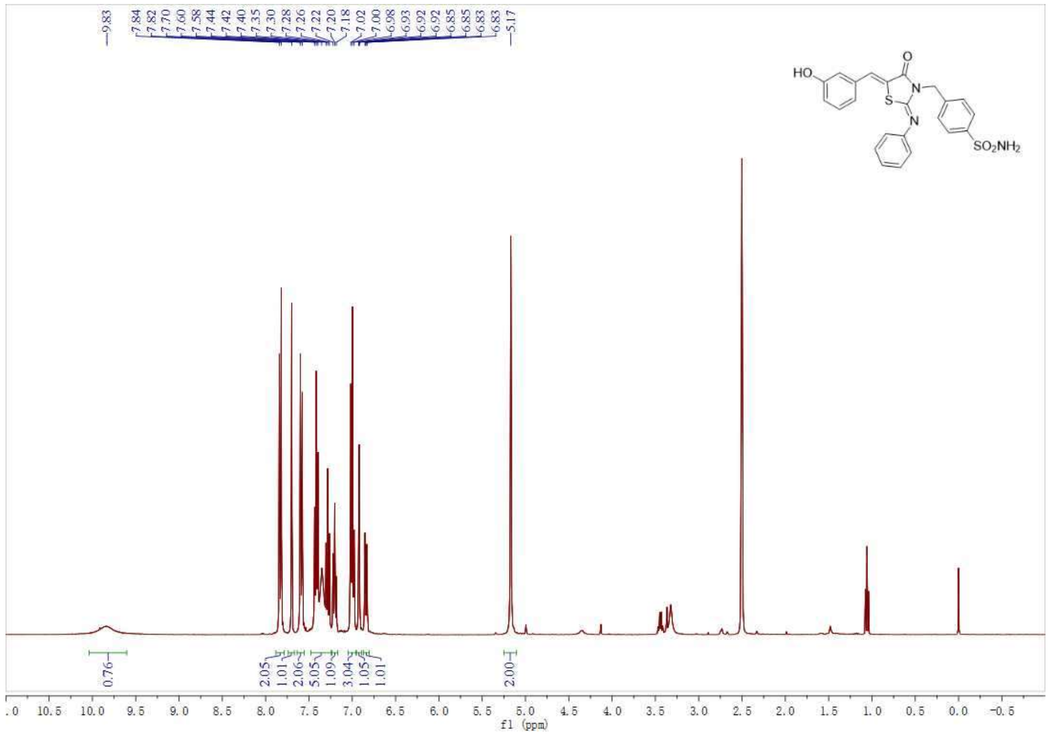

Figure S13. The ^{1}H-NMR of compound 8

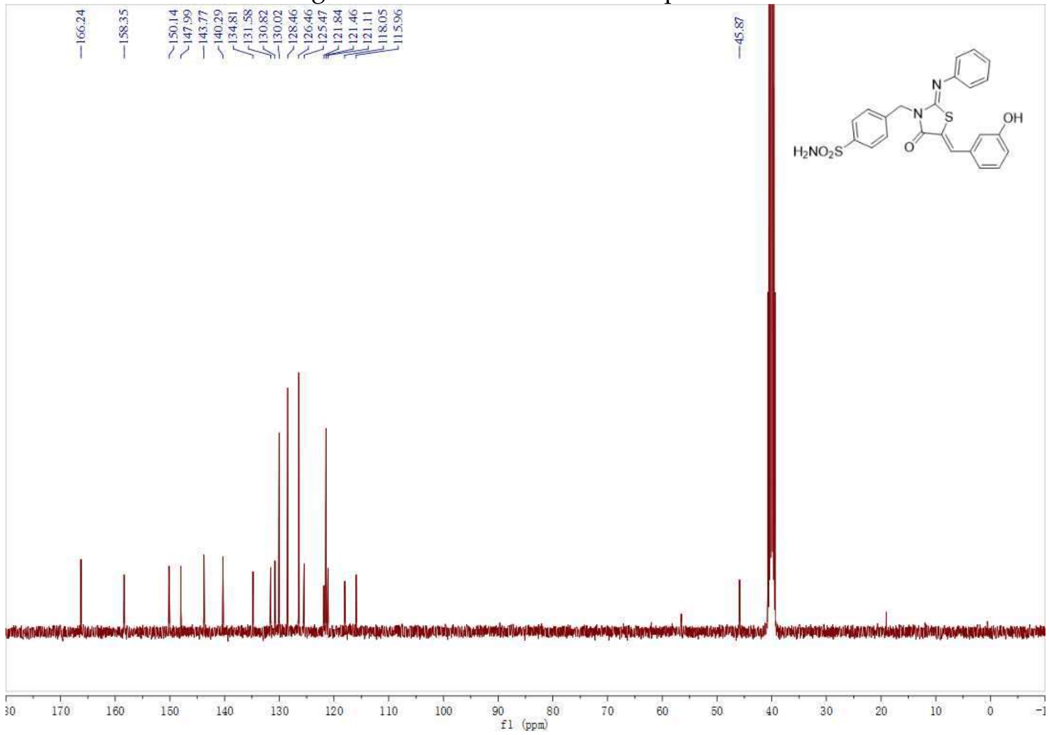

Figure S14. The ^{13}C-NMR of compound 8

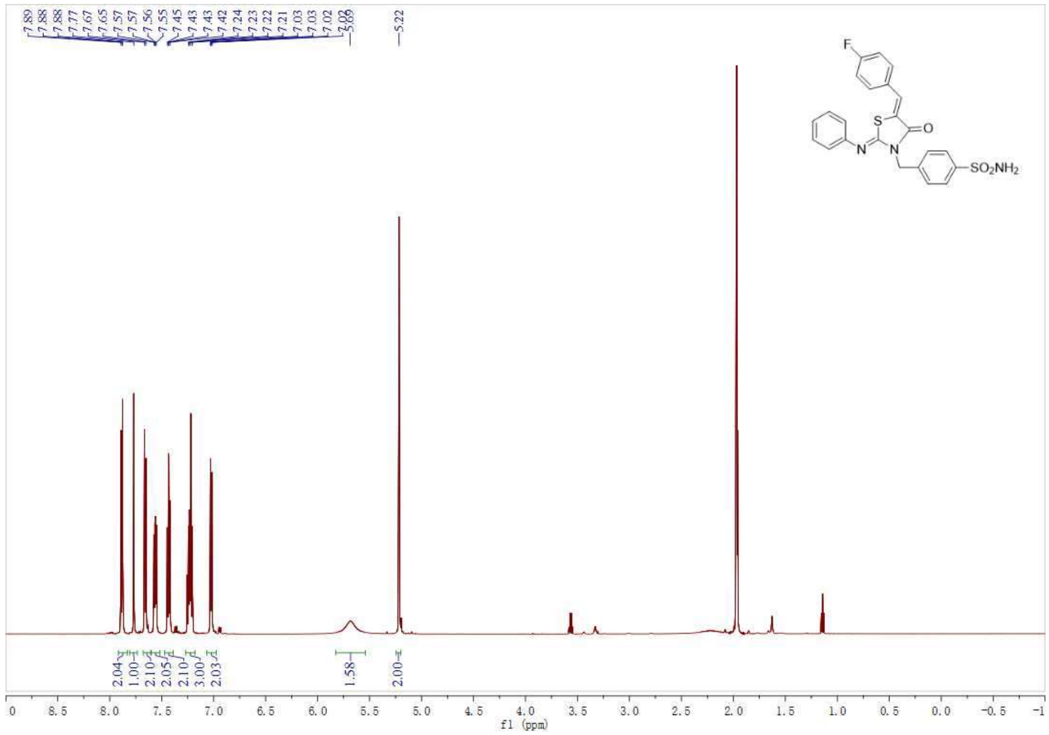

Figure S15. The ^{1}H-NMR of compound 9

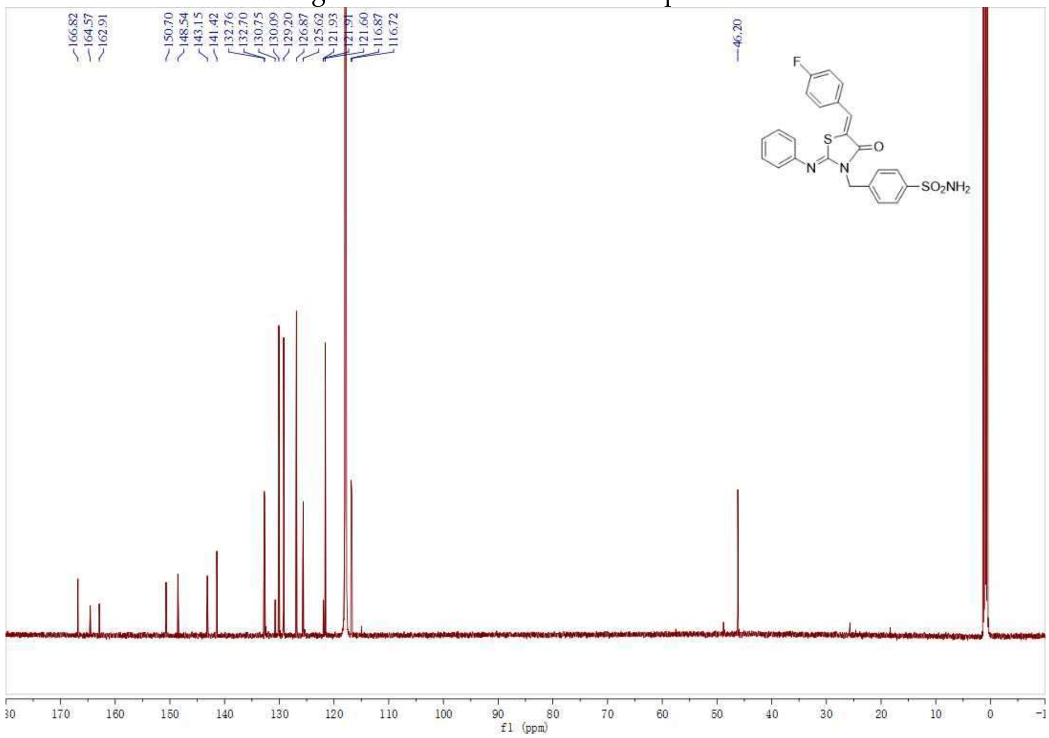

Figure S16. The ^{13}C-NMR of compound 9

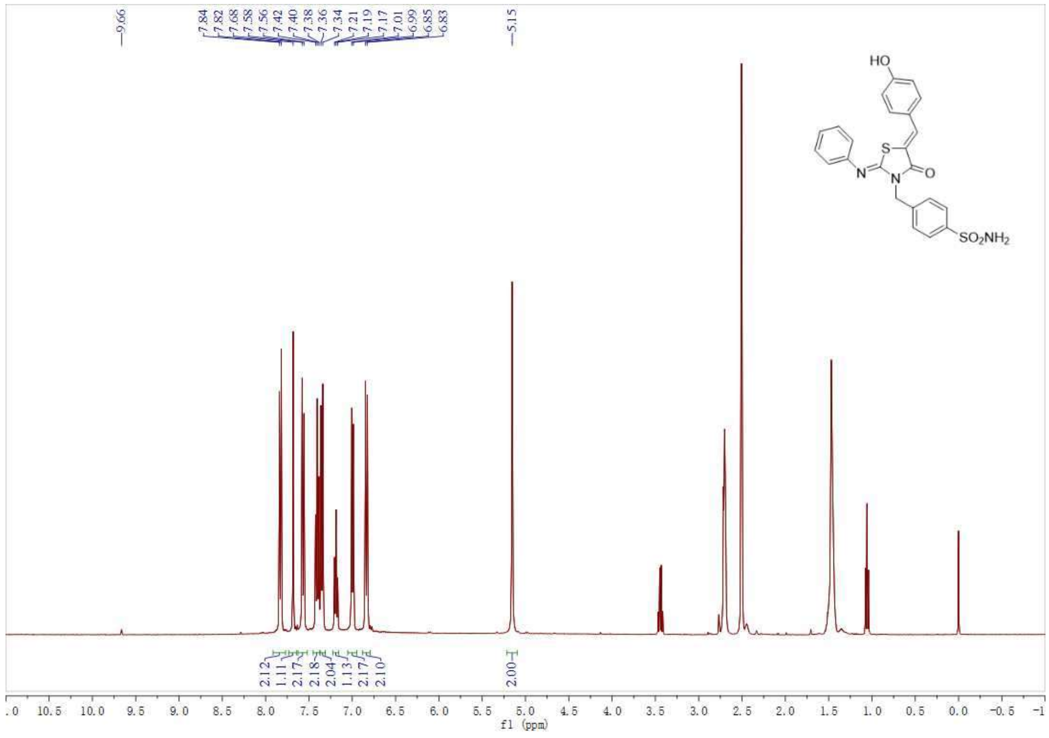

Figure S17. The ^{1}H-NMR of compound 10

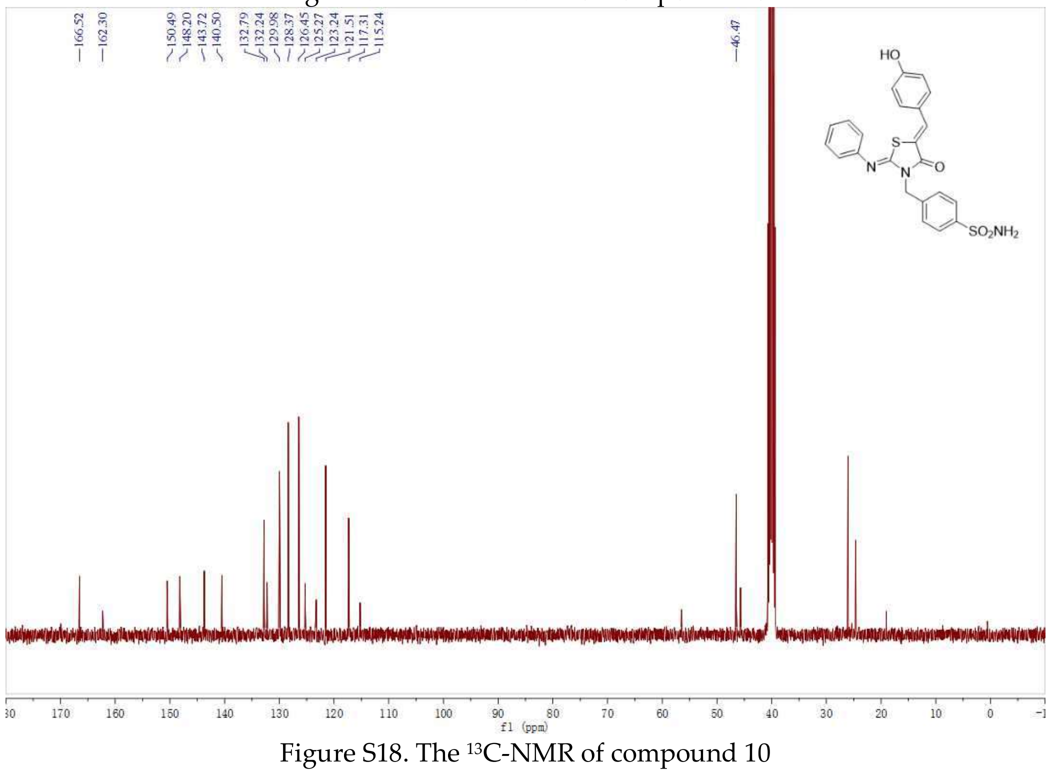

Figure S18. The ^{13}C-NMR of compound 10

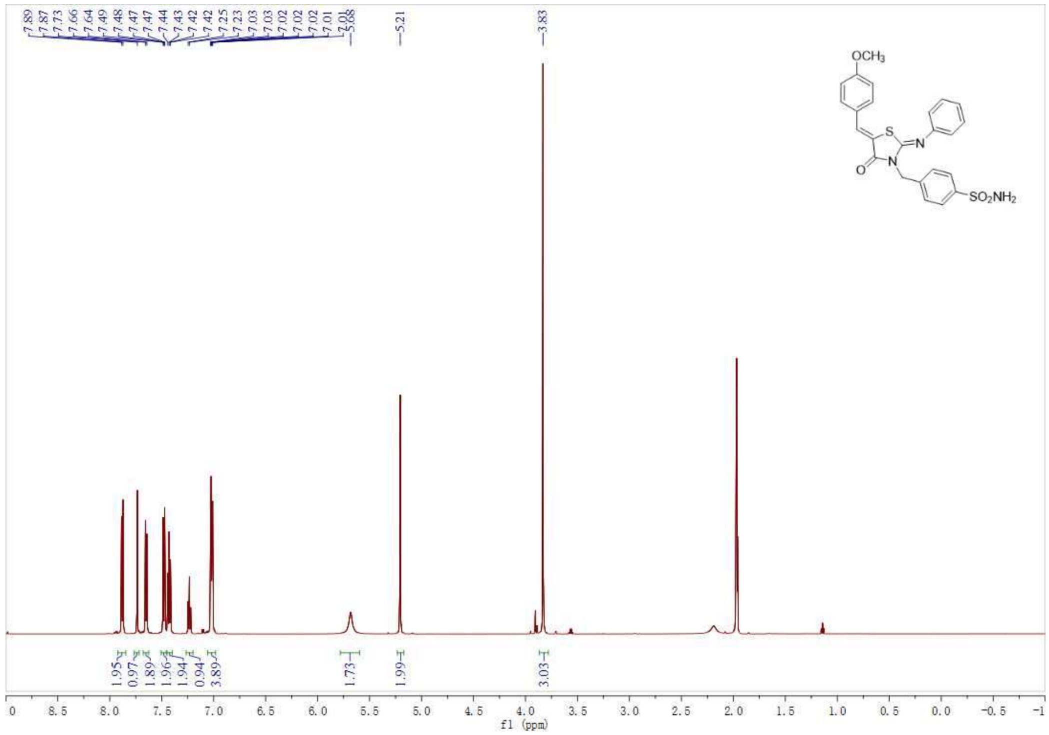

Figure S19. The ^{1}H-NMR of compound 11

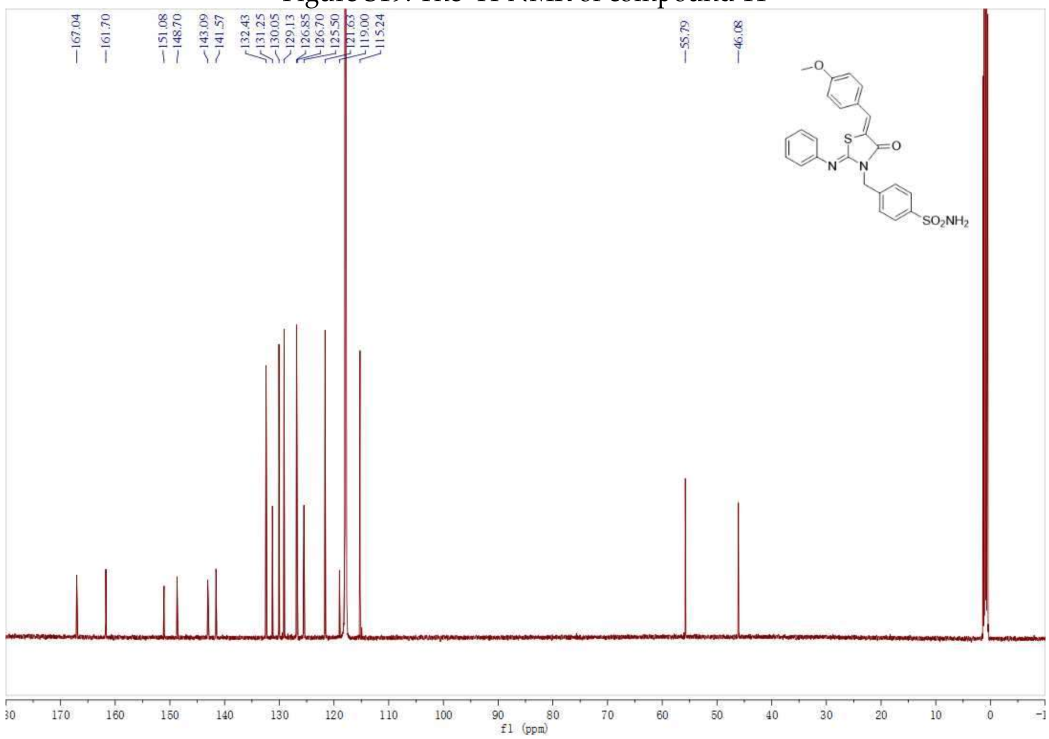

Figure S20. The ^{13}C-NMR of compound 11

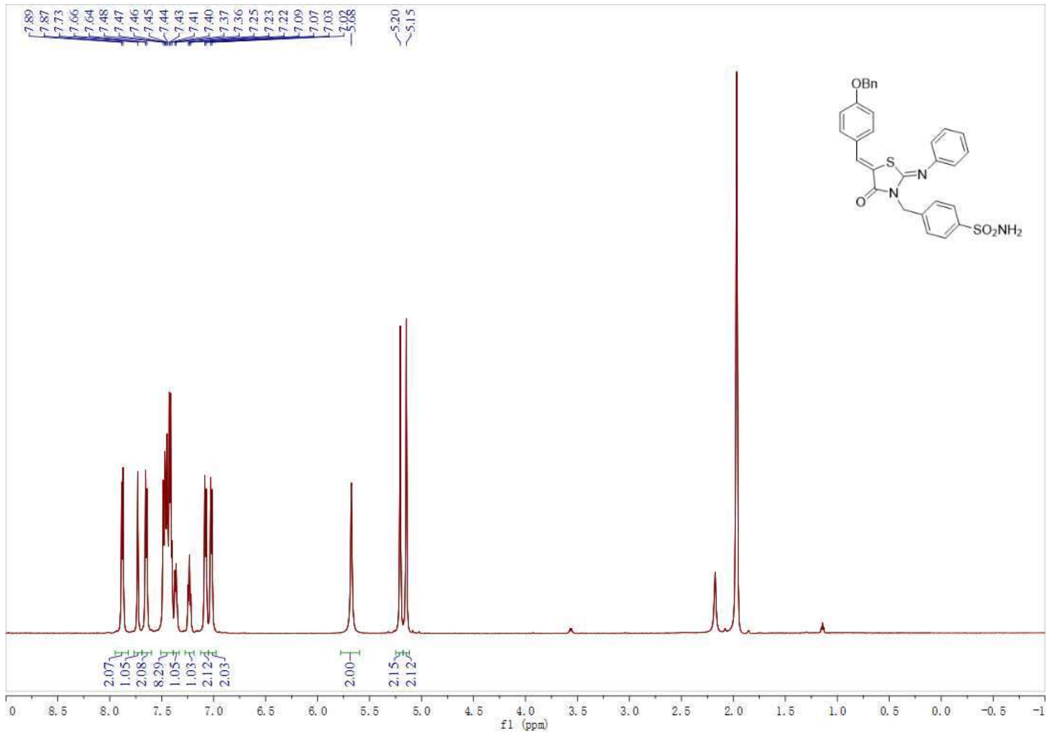

Figure S21. The ^{1}H-NMR of compound 12

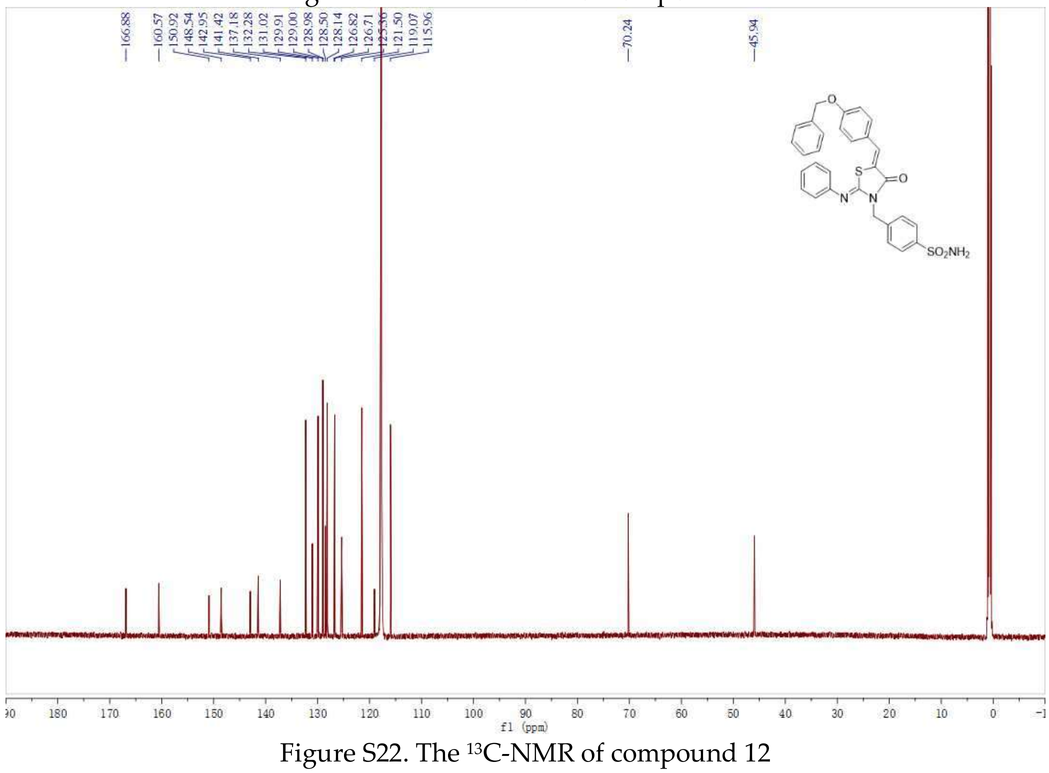

Figure S22. The ^{13}C-NMR of compound 12

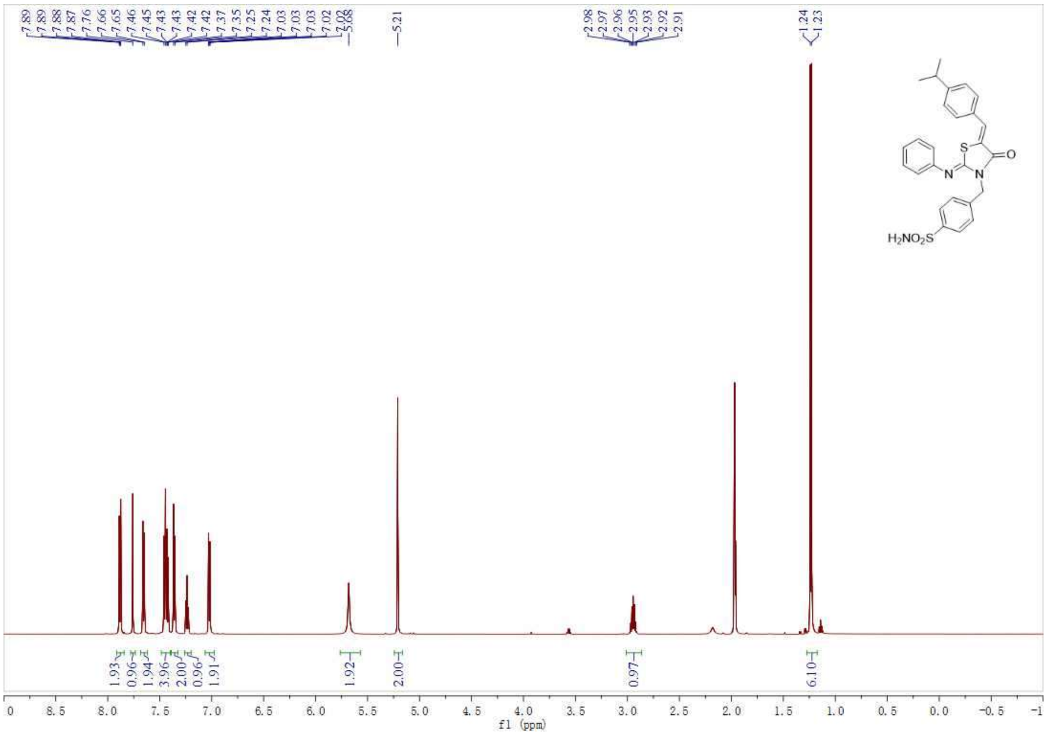

Figure S23. The ^{1}H-NMR of compound 13

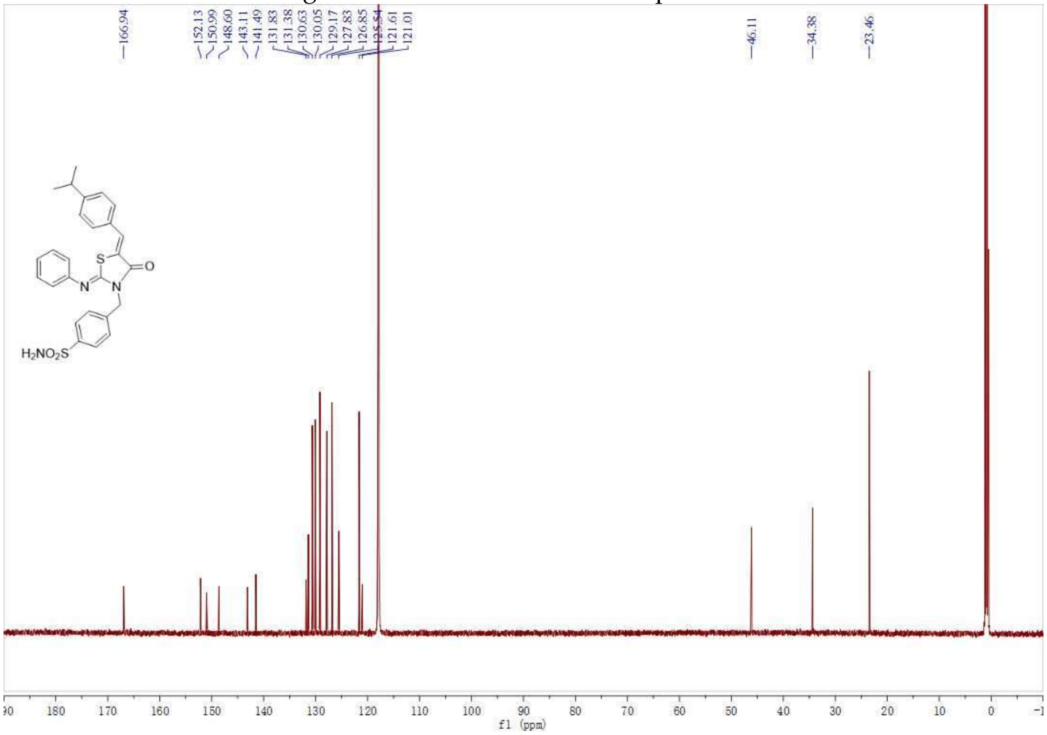

Figure S24. The ^{13}C-NMR of compound 13

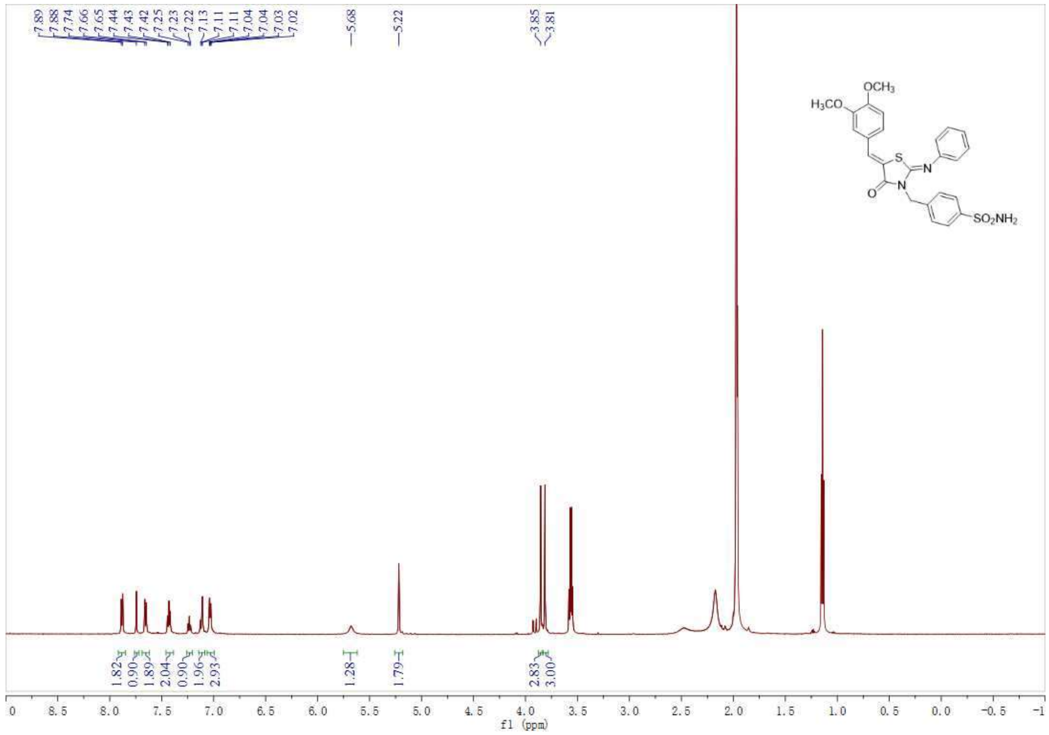

Figure S25. The ^{1}H-NMR of compound 14

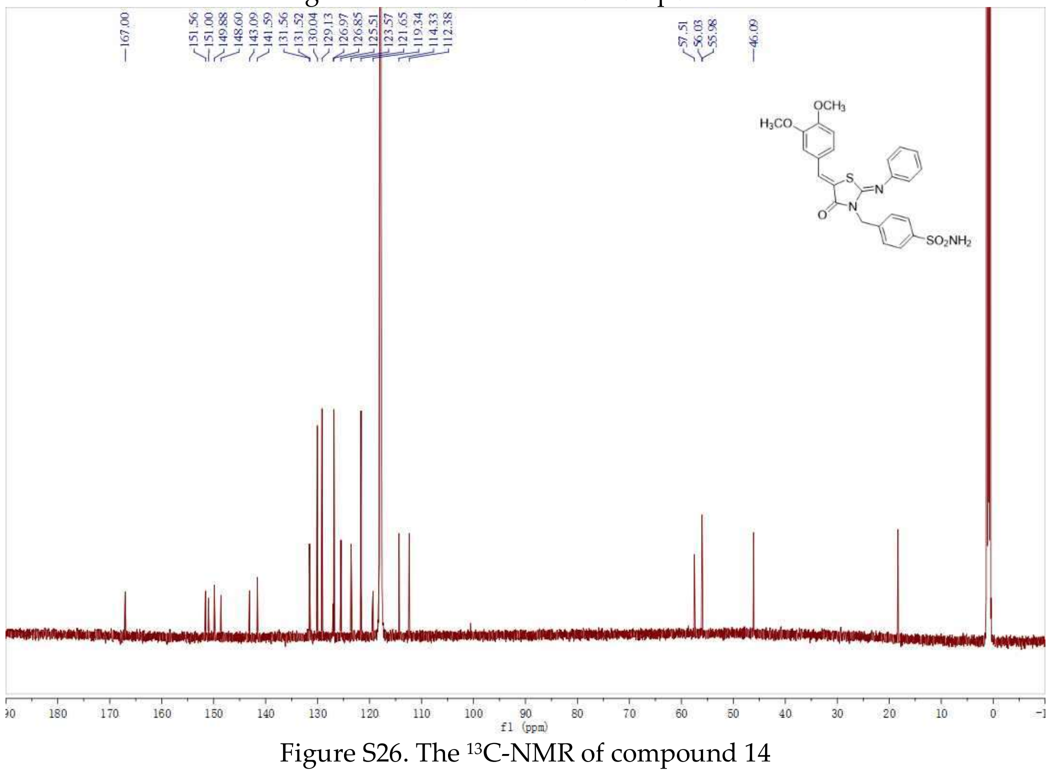

Figure S26. The ^{13}C-NMR of compound 14
